# Isolating Sperm from Cell Mixtures Using Magnetic Beads Coupled with an Anti-PH-20 Antibody for Forensic DNA Analysis

**DOI:** 10.1371/journal.pone.0159401

**Published:** 2016-07-21

**Authors:** Xing-Chun Zhao, Le Wang, Jing Sun, Bo-Wei Jiang, Er-Li Zhang, Jian Ye

**Affiliations:** 1 Beijing Engineering Research Center of Crime Scene Evidence Examination, Institute of Forensic Science, Ministry of Public Security, Beijing, People's Republic of China; 2 Key Laboratory of Forensic Genetics, Institute of Forensic Science, Ministry of Public Security, Beijing, People's Republic of China; 3 Department of Scientific Instruments, the First Research Institute of the Ministry of Public Security, Beijing, People's Republic of China; 4 Department of Criminalistics, People's Public Security University of China, Beijing, People's Republic of China; Duke University Marine Laboratory, UNITED STATES

## Abstract

Vaginal swabs taken in rape cases usually contain epithelial cells from the victim and sperm from the assailant and forensic DNA analysis requires separation of sperm from these cell mixtures. PH-20, which is a glycosylphosphatidylinositol-anchored hyaluronidase located on the head of sperm, has important functions in fertilization. Here we describe a newly developed method for sperm isolation using anti-PH-20 antibody-coupled immunomagnetic beads (anti-PH-20 IMBs). Optical microscopy and scanning electron microscopy showed the IMBs recognized the head of sperm specifically and exhibited a great capacity to capture sperm cells. However, we found it necessary to incubate the IMB–sperm complex with DNase I before sperm lysis in order to remove any female DNA completely. We compared the sensitivity of anti-PH-20 IMBs in sperm and epithelial cell discrimination to those coated with a different anti-sperm antibody (anti-SP-10, anti-ADAM2 or anti-JLP). Only the anti-PH-20 IMBs succeeded in isolating sperm from cell mixtures at a sperm/epithelial cell ratio of 10^3^:10^5^. Further, our method exhibited greater power and better stability for sperm isolation compared to the traditional differential lysis strategy. Taken together, the anti-PH-20 IMB method described here could be effective for the isolation of sperm needed to obtain a single-sourced DNA profile as an aid to identifying the perpetrator in sexual assault cases.

## Introduction

Vaginal swabs taken in rape cases usually contain a mixture of epithelial cells from the victim and sperm from the assailant. In order to obtain the perpetrator’s DNA profile from such cell mixtures, the forensic DNA community has been working on separating the male and female cells. The traditional method for this purpose is differential lysis [1, 2], which involves two kinds of lysis buffer. The first buffer without dithiothreitol (DTT) is used to lyse the epithelial cells preferentially and the second buffer containing DTT can break the protein disulfide bridges responsible for the structure of the sperm nuclear membrane. Due to the variable amounts of cells and the variable sperm/epithelial cell ratios in different vaginal swabs, however, sperm DNA can be released during the first stage due to over-digestion or the epithelial cells might not be broken open totally because of incomplete lysis, resulting in inefficient separation of the male and female fractions [3]. For the past decade, laser capture microdissection (LCM) has been used for capturing sperm directly from cell mixtures obtained by vaginal swabs [3–7]. As stated by Butler, “by physically separating the perpetrator's sperm cells from the victim's epithelial cells, the perpetrator's DNA can be enriched and isolated from even a vast preponderance of victim's cells” [8]. This method is time-consuming, requires expensive instruments and skilled workers, however, which make it unsuitable for use as a routine method. Immunomagnetic beads (IMBs), which are widely used for detecting various tumor cells in clinical investigations [9, 10], have been shown recently to provide an alternative approach for sperm isolation using antibodies against sperm-specific proteins. Anslinger et al. established the first link between IMB technology and the isolation of sperm from cell mixtures obtained by vaginal swabs [11]. Using an antibody against the testicular angiotensin-converting enzyme (tACE), their IMB method yielded mixed DNA profiles for 10^3^–10^5^ sperm cells/mL samples with a major male component in the 10^5^/mL sample. Similarly, the IMBs prepared by Li et al. with an antibody against the motile sperm domain-containing protein 3 (MOSPD3) required the concentration of sperm to be as high as 10^5^ cells/mL for obtaining a full DNA profile of the male donor [12]. To improve the sensitivity and stability of sperm isolation, we prepared magnetic beads coated with the anti-PH-20 antibody. PH-20 (also known as sperm adhesion molecule 1 (SPAM1)) is a glycosylphosphatidylinositol-anchored sperm hyaluronidase enabling acrosome-intact sperm to reach the egg zona pellucida during fertilization [13, 14]. The anti-PH-20 IMB bound to the sperm head specifically and exhibited great sensitivity for sperm isolation from cell mixtures at a sperm/epithelial cell ratio of 10^3^:10^5^. Following incubation of the IMB–sperm complex with DNase I, capillary electrophoresis yielded a single-sourced short tandem repeat (STR) profile of the male donor. Our parallel experiments using simulated sexual assault cell mixtures revealed the anti-PH-20 IMB method is more effective compared to the use of IMB prepared with the antibody against protein SP-10 (sperm protein-10) [15], ADAM2 (a distintegrin and metalloprotease 2) [16, 17] or JLP (JNK-associated leucine zipper protein) [18]. The new anti-PH-20 IMB method provides an alternative solution to the traditional differential lysis approach for genotyping sexual assault cell mixtures.

## Materials and Methods

### Sampling

Simulated sexual assault cell mixtures were prepared with sperm and female epithelial cells donated by unrelated anonymous healthy volunteers. Altogether eight sperm samples, four buccal epithelial cell samples and one vaginal epithelial cell sample were used in this work. Each donor gave written informed consent and this work was approved by the Ethical Review Board of the Institute of Forensic Science, Ministry of Public Security of China. Buccal swabs were incubated in reaction buffer (10 mmol/L PO_4_^3–^, 137 mmol/L NaCl, 2.7 mmol/L KCl, pH 7.4 (PBS) containing 0.1% (w/v) bovine serum albumin) at room temperature for 60 min and semen was diluted tenfold with reaction buffer before the cell concentration was estimated using a Neubauer cell counting chamber. Mixed cell suspensions with known concentrations of cells were prepared with the donated sperm and female cells.

### Preparation of IMB

Dynabeads^®^ M-270 Carboxylic Acid (Invitrogen, Carlsbad, USA) were activated by treatment with 3-(ethyliminomethyleneamino)-*N*,*N*-dimethyl-propan-1-amine (EDC) and *N*-hydroxysuccinimide (NHS) according to the manufacturer’s recommendations. A 60 μg sample of antibody (anti-PH-20, anti-SP-10, anti-ADAM2 or anti-JLP; Abcam, Cambridge, UK) was added to 3 mg of activated beads dissolved in 25 mmol/L 2-(*N*-morpholino)ethanesulfonic acid (MES), pH 5.0. The magnetic beads and antibody mixture was vortex mixed thoroughly and then incubated for 60 min at room temperature with slow tilt rotation. After incubation, the mixture was transferred to a magnet (Promega, Madison, WI). The beads were sufficiently collected in 2 min before the supernatant was removed. The beads were then incubated in 50 mmol/L Tris (pH 7.4) for 15 min to block the unreacted carboxylic acid groups, washed three times with reaction buffer (10 mmol/L PBS, pH 7.4, 0.1% (w/v) bovine serum albumin) containing 0.1% (v/v) Tween-20 and suspended in 100 μL of reaction buffer.

### Sperm isolation

A 10 μL sample of IMBs was mixed with 100 μL of cell mixture and incubated at room temperature for 90 min with slight tilting and rotation before transfer to a magnet for biomagnetic separation. The supernatant was discarded and the IMB–sperm complexes were washed three times with 500 μL of reaction buffer and then treated with an RNase-free DNase Kit (Tiandz, Beijing, China) to remove any attached female DNA according to the manufacturer's recommendation. In detail, 1 μL of reaction buffer, 7 μL of ddH_2_O and 1 μL of DNase I were mixed and incubated with the IMB–sperm complexes at 37°C for 30 min before the digestion reaction was terminated by adding 1 μL of 50 mM EDTA to the reaction mixture and incubating at 65°C for 10 min.

### DNA extraction, amplification and electrophoresis

The IMB–sperm complexes were subjected to DNA extraction procedures using the MagAttract M48 DNA Manual Kit (Qiagen, Hilden, Germany). Extracted DNA was quantified with the Qubit^®^ dsDNA BR Assay Kit (Invitrogen) on a Qubit^®^ fluorometer (Invitrogen) and amplified using the AmpFℓSTR^®^ Identifiler^®^ Plus PCR Amplification Kit (Applied Biosystems, Foster City, USA) on a GeneAmp^®^ 9700 thermal cycler (Applied Biosystems) with 1 ng of DNA in a total reaction volume of 25 μL. Pre-PCR denaturation occurred at 95°C for 11 min. This was followed by 30 cycles of denaturing at 94°C for 1 min, annealing at 59°C for 1 min, extension at 72°C for 1 min and a final extension step at 60°C for 60 min. A 1 μL sample of PCR products was added to 10 μL of deionized formamide containing an internal size standard. All samples were separated on a 3130XL Genetic Analyzer (Applied Biosystems) using POP^™^-4 polymer (Applied Biosystems) and a 36 cm capillary array (Applied Biosystems). Initial fragment sizing and allele calling were done with GeneMapper^®^ ID v3.2 software (Applied Biosystems) with the peak amplitude threshold set at 50 relative fluorescence units for all colors.

## Results

### Anti-PH-20 IMBs bind to the head of sperm

**Fig 1:**
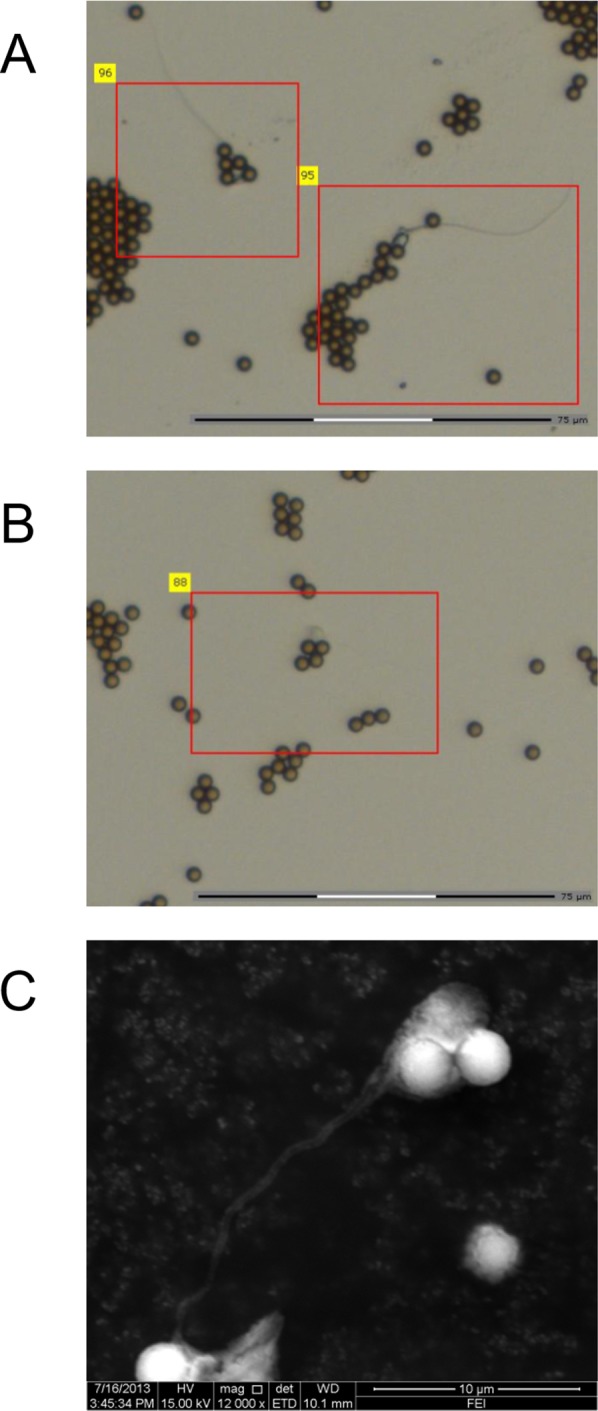
Sperm cells captured by anti-PH-20 IMB. (A) Optical microscopy image for IMB bound to the head of intact sperm cells. (B) Optical microscopy image for IMB bound to the sperm without midpiece and tail. (C) Scanning electron microscopy image for IMB bound to the head of an intact sperm cell. Scale bars are indicated in each panel.

The sperm head contains the nuclear DNA used for STR typing. Therefore, antibodies that recognize the sperm head specifically should be considered a priority for sperm capture. Earlier transmission electron microscopy and immunogold labeling experiments showed PH-20 was located on the plasma membrane over the entire head of human sperm, but not on the midpiece or tail sections [13, 14], which make it a perfect target for isolating sperm with nuclear DNA from cell mixtures. To establish such a method for biomagnetic sperm isolation, an anti-PH-20 antibody was immobilized on commercial magnetic beads with covalent amide bonds and incubated with a simulated cell mixture containing equal concentrations (10^5^/mL) of sperm and female epithelial cells. The beads were washed extensively and then suspended for microscopic observation. Optical microscopy and scanning electron microscopy confirmed the IMB was bound specifically to the heads of both intact (Fig 1A and 1C) and untailed sperm (Fig 1B) and no epithelial cell was found in the suspension.

### DNase-treated IMB–sperm complexes yield single-sourced STR profiles

**Fig 2:**
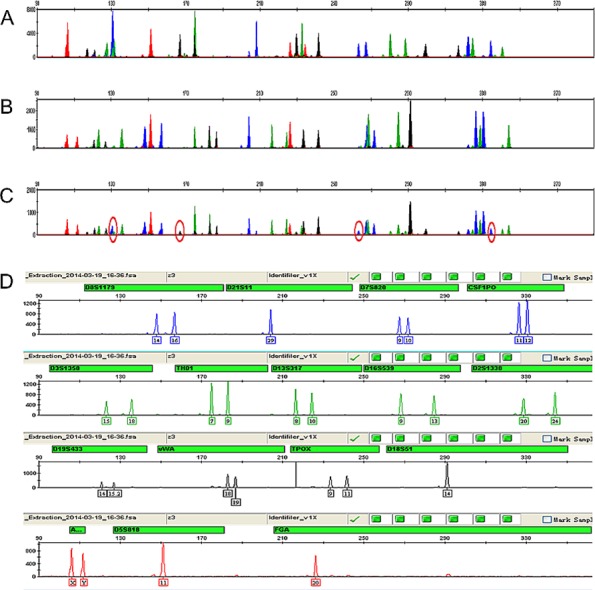
Treatment with DNase I yields a single-sourced DNA profile. (A–C) Electrophoretogram of (A) female epithelial cells, (B) sperm and (C) the IMB–sperm complexes before treatment with DNase I with red circles indicating female DNA contamination. Female epithelial cell and sperm concentrations were both 10^5^/mL. (D) Single-sourced DNA profile of the IMB–sperm complexes after treatment with DNase I.

To examine whether the male fraction has been isolated efficiently, the IMB–sperm complexes were subjected to DNA extraction and STR genotyping. Although epithelial cells were not observed by microscopy (Fig 1), the genotyping experiment yielded a mixed DNA profile (Fig 2C) with the male donor (Fig 2B) as the major component and the female donor (Fig 2A) as the minor component, which is in accord with results reported by others [11]. After extensive review of the experimental procedures we speculated that some female epithelial cells might have been broken before they were separated from the sperm, and the released female DNA could become attached to the surface of the IMB–sperm complexes, leading to a mixed profile in subsequent genotyping. To validate this hypothesis, the IMB–sperm complexes were incubated with DNase I to digest the DNA outside sperm before sperm lysis and DNA extraction. Excitingly, the DNase-treated IMB–sperm complexes gave a single-sourced STR profile of the male donor (Fig 2D), which confirmed our hypothesis.

### Anti-PH-20 IMB is more sensitive than IMBs prepared with a different antibody

To evaluate the efficiency and sensitivity of anti-PH-20 IMBs for isolating sperm, we fixed the epithelial cell concentration at 10^5^/mL and prepared a series of cell mixtures with sperm concentrations ranging from 10^2^–10^5^/mL. At each concentration of sperm, ten simulated vaginal cell mixtures were prepared with cells from different donors. All cell mixtures at sperm concentrations of 10^5^/mL and 10^4^/mL were fractionated successfully and capillary electrophoresis yielded single-sourced DNA profiles of each male donor (Table 1). At the sperm concentration of 10^3^/mL, sperm from nine cell mixtures were isolated effectively by anti-PH-20 IMBs leaving only one sample not genotyped, whereas no cell mixture yielded single-sourced profile at a sperm concentration of 10^2^/mL due to the extreme epithelial cell to sperm ratio of 1000:1.

**Table 1:** Comparison of the sensitivity of different antibodies in capturing sperm.

Sperm concentration in cell mixture	Number of successfully genotyped single-sourced profiles			
	Anti-PH-20	Anti-SP-10	Anti-ADAM2	Anti-JLP
10^5^/mL	10	10	10	10
10^4^/mL	10	9	7	8
10^3^/mL	9	0	0	0
10^2^/mL	0	0	0	0

Simulated vaginal cell mixtures were prepared by mixing female epithelial cells with sperm at different concentrations (ten mixtures for each concentration).

As well as the anti-PH-20 antibody, antibodies against proteins SP-10 [15], ADAM2 [16, 17] and JLP [18] were coupled to magnetic beads and tested for sensitivity of sperm isolation. According to our parallel experiments, these three types of IMB were able to separate sperm from the mixtures with a sperm concentration of 10^5^/mL or 10^4^/mL but failed when the sperm concentration was 10^3^/mL (Table 1). Their sensitivity for discriminating between sperm and epithelial cells was equivalent to those reported for anti-tACE [11] and anti-MOSPD3 [12] IMBs. The anti-PH-20 IMB exhibited excellent sensitivity compared to other antibodies, and isolated sperm successfully from simulated vaginal cell mixtures at a sperm concentration of 10^3^/mL.

### Comparison of the anti-PH-20 IMB and the differential lysis method

Because our data suggested the use of anti-PH-20 IMBs was a powerful and sensitive method for separating sperm from cell mixtures, we attempted to compare its level of efficiency with the traditional differential lysis strategy. In all, 20 cell mixtures were prepared with the epithelial cell and sperm concentration fixed at 10^5^/mL and 10^3^/mL, respectively, and these mixtures were subjected to anti-PH-20-IMB-based and differential-lysis-based sperm isolation and STR genotyping. The anti-PH-20 IMB method succeeded in producing single-sourced DNA profiles at a proportion of 90% but the differential lysis strategy yielded only one single-sourced profile out of 20 cell mixtures (Table 2 and S1 Fig) due to the extreme ratio between sperm and epithelial cells. These results suggested that anti-PH-20 IMBs might provide an effective solution to the isolation of sperm from sexual assault cell mixtures.

**Table 2:** Comparison between anti-PH-20 IMB and differential lysis.

Sperm isolation strategy	Single-sourced profile of the male donor	Mixed profile
Anti-PH-20 IMB	18	2
Differential lysis	1	19

Cell concentrations were fixed at 10^5^/mL (female epithelial cells) and 10^3^/mL (sperm).

## Discussion

A vaginal swab containing a mixture of cells from the victim and the assailant taken following a sexual assault is one of the most frequently encountered types of biological evidence in forensic DNA laboratories. Unfortunately, the efficiency and success rate for genotyping the male perpetrator as a single-sourced profile have never been satisfactory. The stumbling block is how to separate sperm reliably from the abundant female component in cell mixtures. We describe an effective and sensitive approach for sperm isolation based on the use of anti-PH-20 antibody and magnetic beads, which proved to be capable of separating sperm from a mixture of cells at extreme female/male cell ratios.

IMB-based sperm isolation has been suggested for some time, and at least two types of anti-sperm antibodies (anti-tACE and anti-MOSPD3) have been examined. The anti-tACE antibody was associated with magnetic beads via antigen–antibody interactions [11] and the coupling between the anti-MOSPD3 antibody and magnetic beads was mediated by the avidin–biotin system [12]. In this study, the anti-PH-20 antibody was linked to the carboxylic group of magnetic beads via amide bonds, which guaranteed anti-PH-20 IMB as a stable and inseparable entity during experimental procedures. To our knowledge, this is the first report of an anti-sperm antibody linked to magnetic beads via covalent bonds for sperm isolation from vaginal cell mixtures.

Incomplete separation of the sperm and female cells has been a problem for both the traditional differential lysis method and the newly introduced biomagnetic separation strategy. For example, earlier work based on the anti-tACE antibody yielded single-sourced DNA profiles only for samples with a sperm concentration of 10^6^/mL, but not for those with a sperm concentration of 10^5^/mL or lower [11]. Our work based on the anti-PH-20 IMB (Fig 2) and three other anti-sperm antibodies encountered the same problems, although epithelial cells were not observed by microscopy (Fig 1). We hypothesized that some epithelial cells might be lysed during the experimental manipulations and cellular debris, including female DNA, could become attached to the surface of the complexes. The hypothesis was confirmed by incubating the IMB–sperm with DNase I, which yielded single-sourced STR profiles (Fig 2 and S2 Fig). This technical modification, which worked for the anti-PH-20 IMB and the other three anti-sperm antibodies (Table 1), might provide further clues for solving similar problems for forensic DNA as well as clinical diagnosis.

Our method based on anti-PH-20 IMBs successfully isolated sperm at a concentration of 10^3^/mL from cell mixtures containing female epithelial cells at a concentration of 10^5^/mL, exhibiting advantageous sensitivity over anti-SP-10, anti-ADAM2 and anti-JLP antibodies (Table 1) as well as published work [11, 12]. One important reason for the satisfactory sensitivity is that the localization of the PH-20 protein fulfills our expectations for a sperm-capturing target. STR genotyping requires nuclear DNA as templates for PCR. As the nuclear DNA of a sperm is located inside the sperm head, but not in the midpiece or tail part, the proteins located on the surface of sperm heads should be preferentially considered as targets for sperm capture. The PH-20 protein was present on the plasma membrane over the entire head of human sperm, enabling the IMB prepared with its antibody to capture both the intact and untailed sperm (Fig 1). Earlier immunohistochemical staining studies showed anti-tACE IMBs failed to capture some sperm without flagellum and midpiece, which is consistent with the fact that the tACE protein was localized only on the post-acrosomal area, neck and midpiece of the sperm [11]. In this study, anti-SP-10 IMB showed inferior sensitivity compared to the anti-PH-20 IMB, and the difference is likely because the SP-10 protein is present only at the equatorial region of the sperm head [15].

Remarkably, our biomagnetic sperm separation method based on the anti-PH-20 antibody had greater sensitivity compared to the differential lysis approach (Table 2). It is worth noting these results were obtained with simulated cell mixtures. The performance of the anti-PH-20 IMB procedure with biological evidence from real cases requires further evaluation and optimization.

Besides forensic DNA applications, sperm selection with magnetic beads is also one of the advanced strategies for assisted reproduction. For example, Annexin V-conjugated paramagnetic microbeads have been used to eliminate apoptotic spermatozoa from sperm suspensions so that a fraction of spermatozoa with normal morphology and less DNA damage can be prepared [19, 20]. Our work based on the anti-PH-20 IMB might provide further hints in target protein selection, bead-protein conjugation manner selection and experimental procedures for assisted reproduction as well as other applications that work with sperm in complex systems.

In summary, we developed a novel method for sperm isolation from vaginal cell mixtures based on magnetic beads coated with an anti-PH-20 antibody, which is sensitive for cell type discrimination and effective in removing the female fraction completely, providing a promising approach for separating sexual assault cell mixtures.

## Supporting Information

S1 Fig Sperm isolation with the anti-PH20 IMB and differential lysis. (PDF)

S2 Fig Sperm isolation from a mixture of sperm and vaginal epithelial cells using the anti-PH-20 IMB. (PDF)

## References

[1] Gill P, Jeffreys AJ, Werrett DJ. Forensic application of DNA 'fingerprints'. Nature. 1985;318(6046):577–9. 3840867 10.1038/318577a0

[2] Yoshida K, Sekiguchi K, Mizuno N, Kasai K, Sakai I, Sato H, et al The modified method of two-step differential extraction of sperm and vaginal epithelial cell DNA from vaginal fluid mixed with semen. Forensic science international. 1995;72(1):25–33. 7705732 10.1016/0379-0738(94)01668-u

[3] Elliott K, Hill DS, Lambert C, Burroughes TR, Gill P. Use of laser microdissection greatly improves the recovery of DNA from sperm on microscope slides. Forensic science international. 2003;137(1):28–36. 14550610 10.1016/s0379-0738(03)00267-6

[4] Sanders CT, Sanchez N, Ballantyne J, Peterson DA. Laser microdissection separation of pure spermatozoa from epithelial cells for short tandem repeat analysis. Journal of forensic sciences. 2006;51(4):748–57. 16882215 10.1111/j.1556-4029.2006.00180.x

[5] Anslinger K, Bayer B, Mack B, Eisenmenger W. Sex-specific fluorescent labelling of cells for laser microdissection and DNA profiling. International journal of legal medicine. 2007;121(1):54–6. 16552569 10.1007/s00414-005-0065-7

[6] Vandewoestyne M, Van Nieuwerburgh F, Van Hoofstat D, Deforce D. Evaluation of three DNA extraction protocols for forensic STR typing after laser capture microdissection. Forensic science international Genetics. 2012;6(2):258–62. 10.1016/j.fsigen.2011.06.002 21727054

[7] Budimlija ZM, Lechpammer M, Popiolek D, Fogt F, Prinz M, Bieber FR. Forensic applications of laser capture microdissection: use in DNA-based parentage testing and platform validation. Croatian medical journal. 2005;46(4):549–55. 16100757

[8] Butler JM. Chapter 2—DNA Extraction Methods. Advanced Topics in Forensic DNA Typing: Methodology San Diego: Academic Press; 2012 p. 29–47.

[9] Naume B, Borgen E, Kvalheim G, Karesen R, Qvist H, Sauer T, et al Detection of isolated tumor cells in bone marrow in early-stage breast carcinoma patients: comparison with preoperative clinical parameters and primary tumor characteristics. Clinical cancer research: an official journal of the American Association for Cancer Research. 2001;7(12):4122–9. 11751511

[10] Hung LY, Chuang YH, Kuo HT, Wang CH, Hsu KF, Chou CY, et al An integrated microfluidic platform for rapid tumor cell isolation, counting and molecular diagnosis. Biomedical microdevices. 2013;15(2):339–52. 10.1007/s10544-013-9739-y 23315192

[11] Anslinger K, Bayer B, Danilov SM, Metzger R. Application of sperm-specific antibodies for the separation of sperm from cell mixtures. Forensic science international Genetics. 2008;Supplement Series(1):394–5.

[12] Li XB, Wang QS, Feng Y, Ning SH, Miao YY, Wang YQ, et al Magnetic bead-based separation of sperm from buccal epithelial cells using a monoclonal antibody against MOSPD3. International journal of legal medicine. 2014;128(6):905–11. 10.1007/s00414-014-0983-3 24590379

[13] Baba D, Kashiwabara S, Honda A, Yamagata K, Wu Q, Ikawa M, et al Mouse sperm lacking cell surface hyaluronidase PH-20 can pass through the layer of cumulus cells and fertilize the egg. The Journal of biological chemistry. 2002;277(33):30310–4. 12065596 10.1074/jbc.M204596200

[14] Sabeur K, Cherr GN, Yudin AI, Primakoff P, Li MW, Overstreet JW. The PH-20 protein in human spermatozoa. Journal of andrology. 1997;18(2):151–8. 9154509

[15] Hamatani T, Tanabe K, Kamei K, Sakai N, Yamamoto Y, Yoshimura Y. A monoclonal antibody to human SP-10 inhibits in vitro the binding of human sperm to hamster oolemma but not to human Zona pellucida. Biology of reproduction. 2000;62(5):1201–8. 10775167 10.1095/biolreprod62.5.1201

[16] Kim E, Lee JW, Baek DC, Lee SR, Kim MS, Kim SH, et al Processing and subcellular localization of ADAM2 in the Macaca fascicularis testis and sperm. Animal reproduction science. 2010;117(1–2):155–9. 10.1016/j.anireprosci.2009.04.002 19443142

[17] Rosselot C, Kierszenbaum AL, Rivkin E, Tres LL. Chronological gene expression of ADAMs during testicular development: prespermatogonia (gonocytes) express fertilin beta (ADAM2). Developmental dynamics: an official publication of the American Association of Anatomists. 2003;227(3):458–67. 12815633 10.1002/dvdy.10327

[18] Iwanaga A, Wang G, Gantulga D, Sato T, Baljinnyam T, Shimizu K, et al Ablation of the scaffold protein JLP causes reduced fertility in male mice. Transgenic research. 2008;17(6):1045–58. 10.1007/s11248-008-9191-6 18574703

[19] Henkel R. Sperm preparation: state-of-the-art—physiological aspects and application of advanced sperm preparation methods. Asian journal of andrology. 2012;14(2):260–9. 10.1038/aja.2011.133 22138904 PMC3735088

[20] Grunewald S, Paasch U, Said TM, Rasch M, Agarwal A, Glander HJ. Magnetic-activated cell sorting before cryopreservation preserves mitochondrial integrity in human spermatozoa. Cell and tissue banking. 2006;7(2):99–104. 16732412 10.1007/s10561-005-1367-1

