# Supplementary material for: Isolating Sperm from Cell Mixtures Using Magnetic Beads Coupled with an Anti-PH-20 Antibody for Forensic DNA Analysis
Source: PLoS One. 2016 Jul 21;11(7):e0159401. doi: 10.1371/journal.pone.0159401 (PMC4956189; doi:10.1371/journal.pone.0159401)
Supplement: S1 Fig — (PDF) [file pone.0159401.s001.pdf]

A

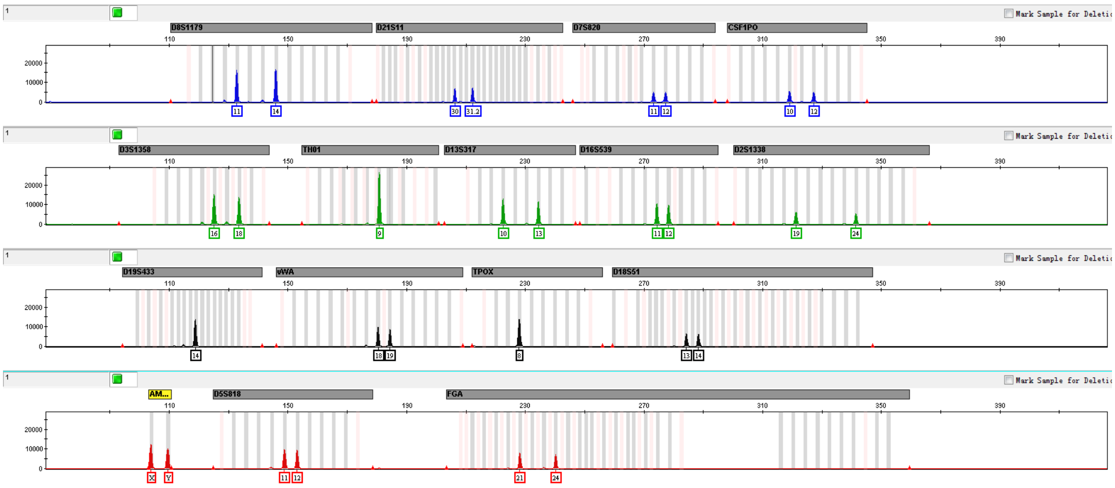
D8S1179
D21S11
D7S820
CSF1PO
D3S1358
TH01
D13S317
D16S539
D2S1338
D19S433
vWA
TPOX
D18S51
AM...
D5S818
FGA

B

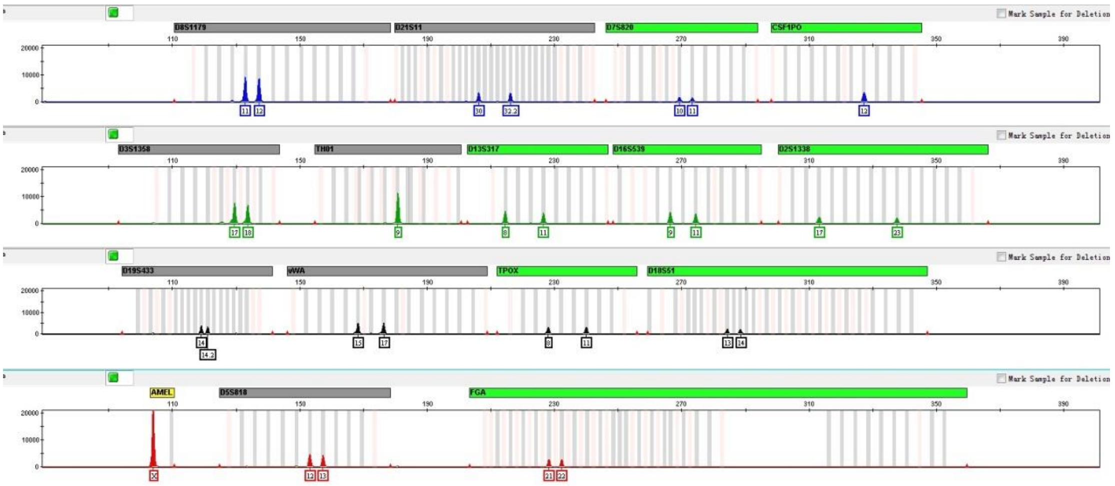
D8S1179
D21S11
D7S820
CSF1PO
D3S1358
TH01
D13S317
D16S539
D2S1338
D19S433
vWA
TPOX
D18S51
AMEL
D5S818
FGA
Mark Sample for Deletion

C

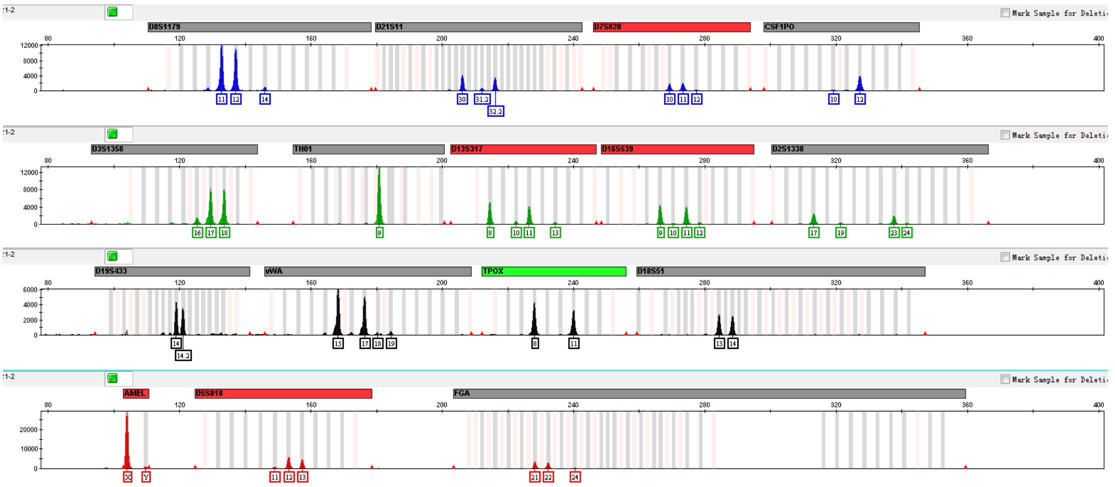
D8S1179
D21S11
D7S820
CSF1PO
D3S1358
TH01
D13S317
D16S539
D2S1338
D19S433
vWA
TPOX
D18S51
AMEL
D5S818
FGA

D

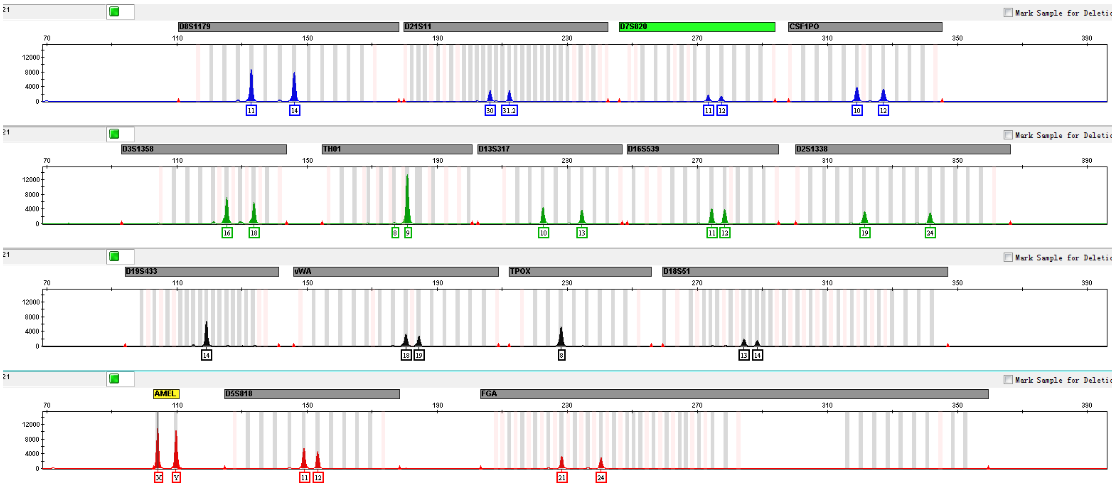

Mark Sample for Deletion
D8S1179
D21S11
D7S820
CSF1PO
11 14 30 31.2 11 12 10 12
D3S1358
TH01
D13S317
D16S539
D2S1338
16 18 8 9 10 13 11 12 19 24
D19S433
vWA
TPOX
D18S51
14 18 19 8 13 14
AMEL
D5S818
FGA
X Y 11 12 21 24

E

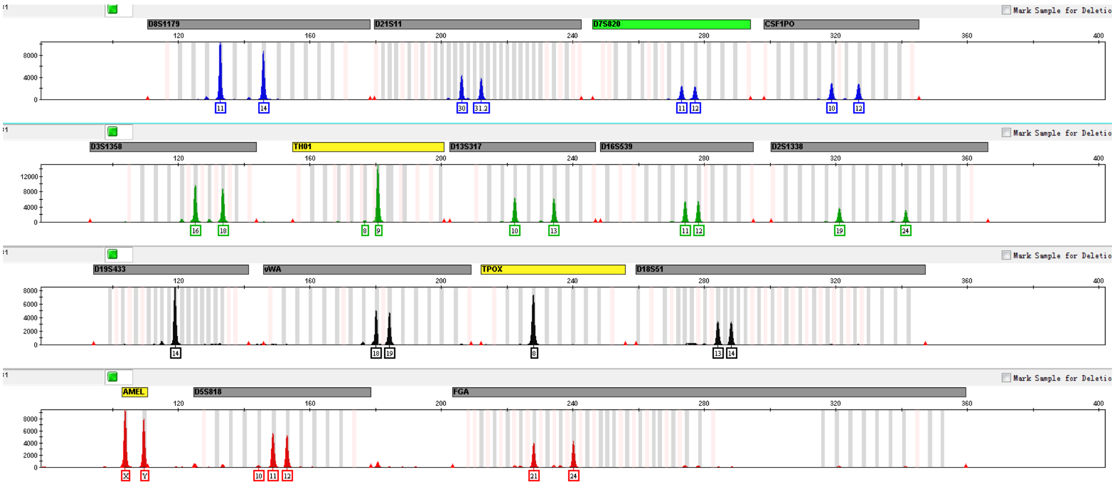

Mark Sample for Deletion
D8S1179
D21S11
D7S820
CSF1PO
11 14 30 31.2 11 12 10 12
D3S1358
TH01
D13S317
D16S539
D2S1338
16 18 8 9 10 13 11 12 19 24
D19S433
vWA
TPOX
D18S51
14 18 19 8 13 14
AMEL
D5S818
FGA
X Y 10 11 12 21 24

F

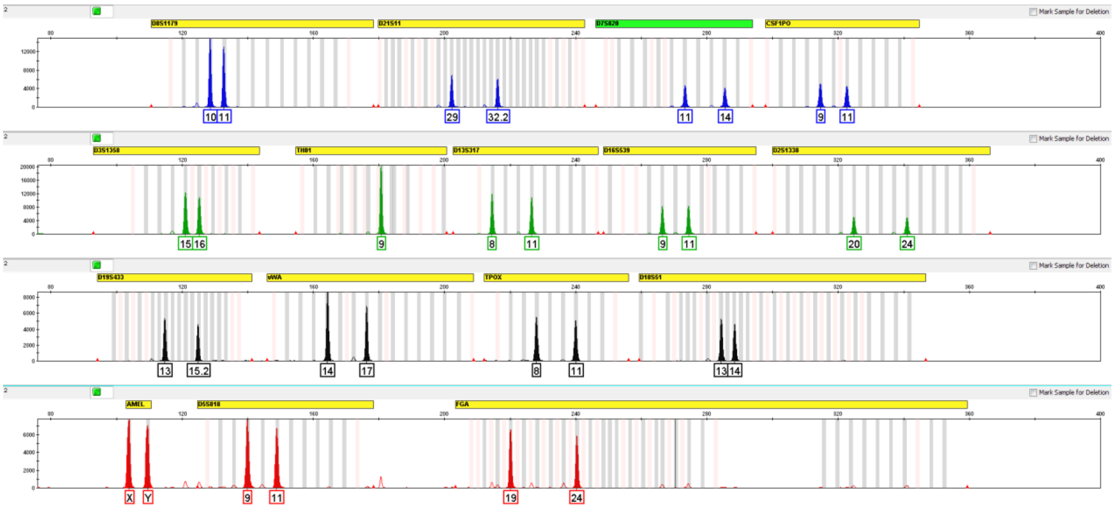

Mark Sample for Deletion
D8S1179
D21S11
D7S820
CSF1PO
10 11 29 32.2 11 14 9 11
D3S1358
TH01
D13S317
D16S539
D2S1338
15 16 9 8 11 9 11 20 24
D19S433
vWA
TPOX
D18S51
13 15.2 14 17 8 11 13 14
AMEL
D5S818
FGA
X Y 9 11 19 24

G

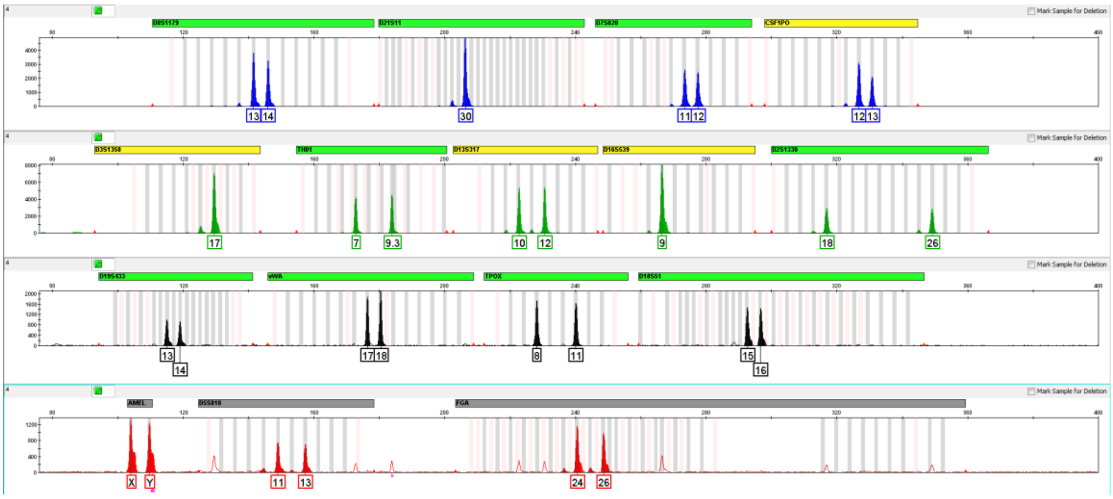
D8S1179
D21S11
D7S820
CSF1PO
13 14
30
11 12
12 13
D3S1358
TH01
D13S317
D16S539
D2S1338
17
7
9.3
10
12
9
18
26
D19S433
vWA
TPOX
D18S51
13
14
17 18
8
11
15
16
AMEL
D5S818
FGA
X
Y
11
13
24
26

H

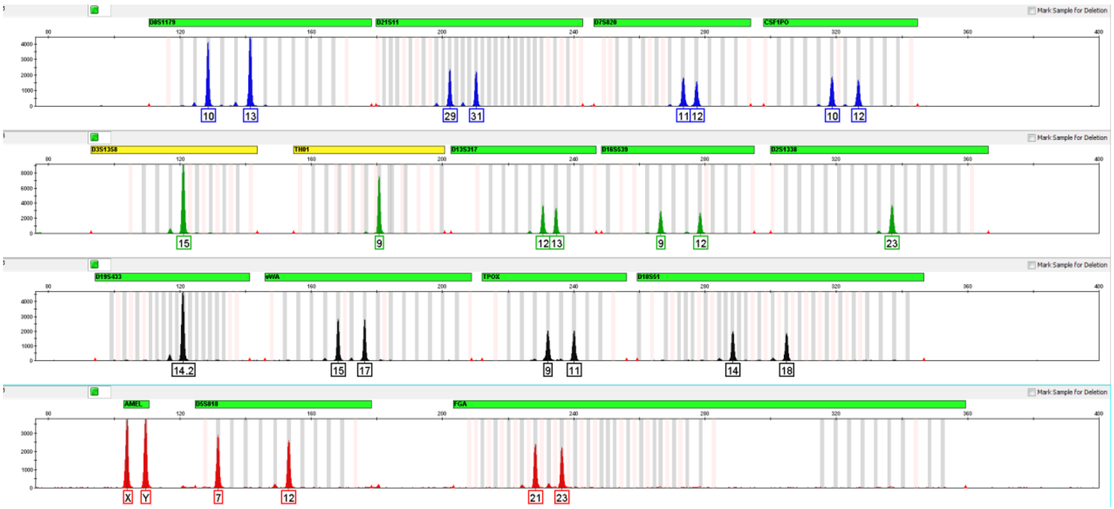
D8S1179
D21S11
D7S820
CSF1PO
10
13
29
31
11 12
10
12
D3S1358
TH01
D13S317
D16S539
D2S1338
15
9
12 13
9
12
23
D19S433
vWA
TPOX
D18S51
14.2
15
17
9
11
14
18
AMEL
D5S818
FGA
X
Y
7
12
21
23

I

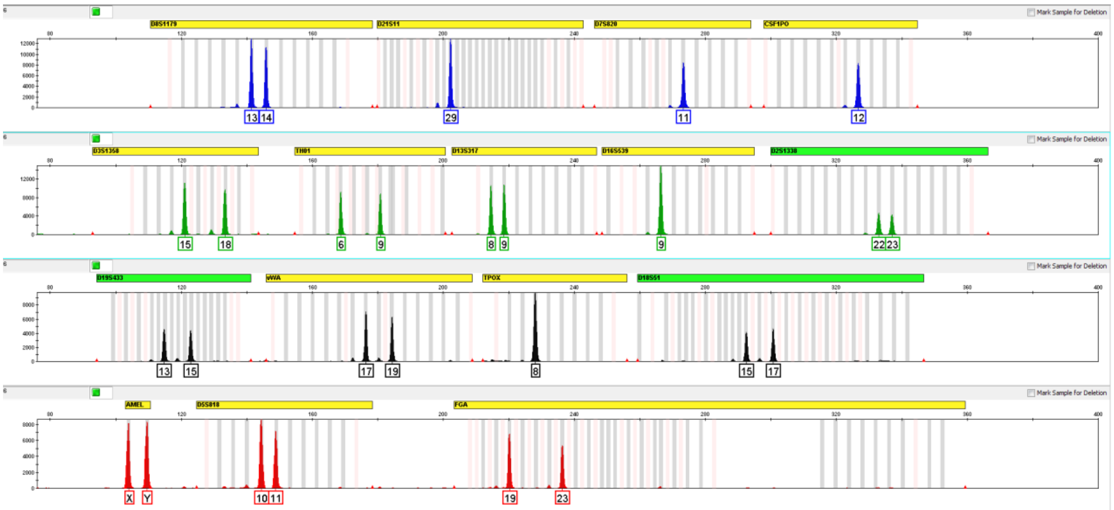
D8S1179
D21S11
D7S820
CSF1PO
13 14
29
11
12
D3S1358
TH01
D13S317
D16S539
D2S1338
15
18
6
9
8
9
9
22 23
D19S433
vWA
TPOX
D18S51
13
15
17
19
8
15
17
AMEL
D5S818
FGA
X
Y
10 11
19
23

J

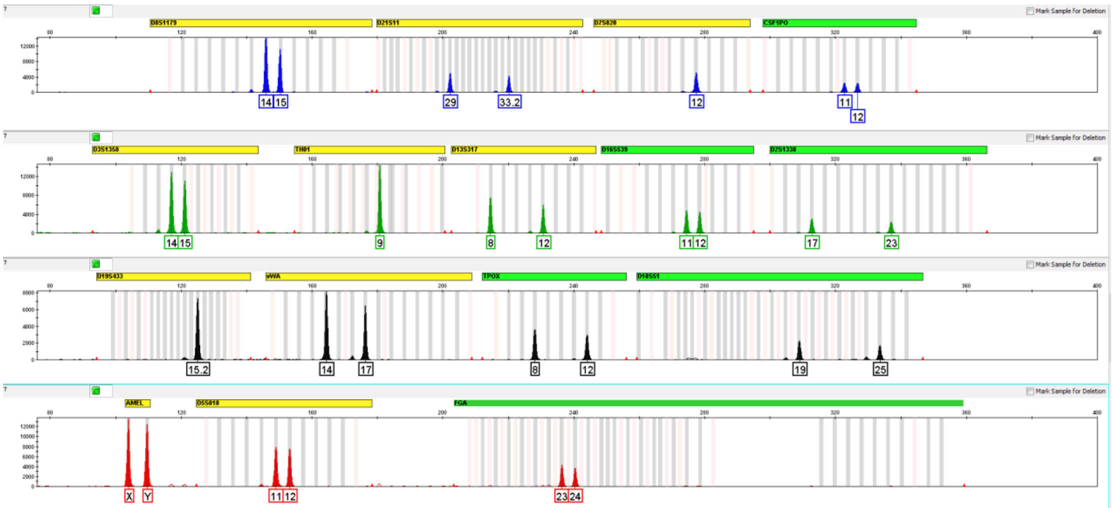

K

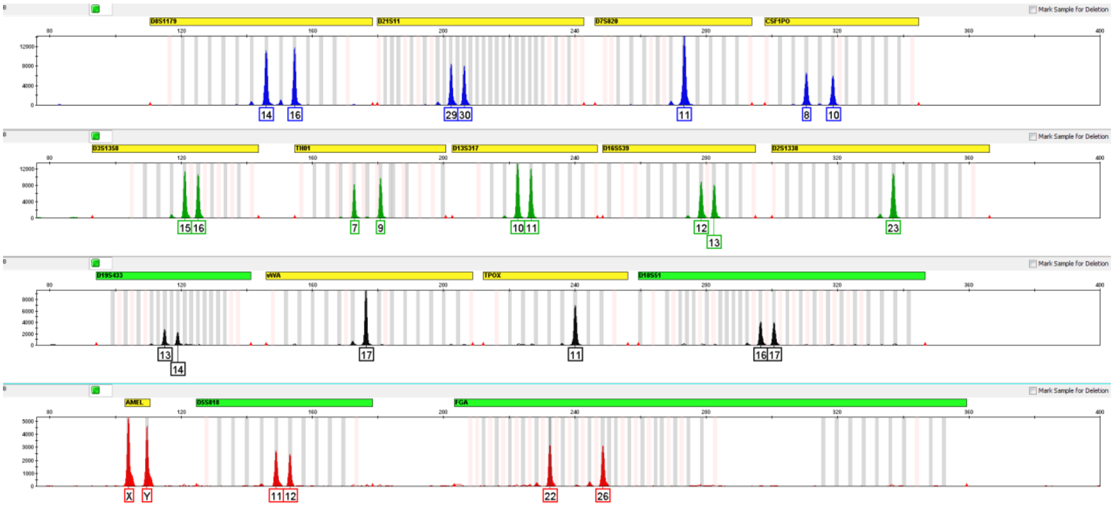

L

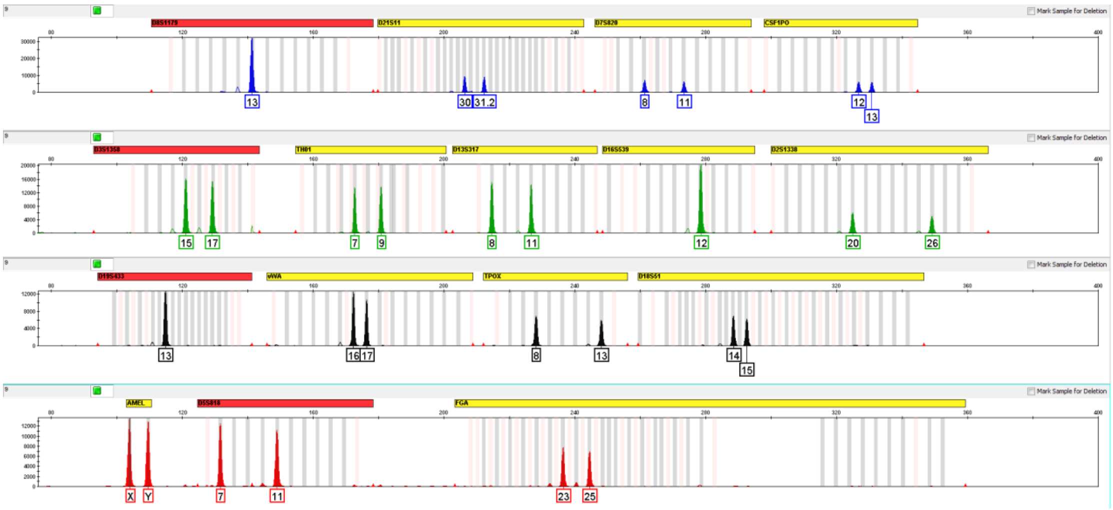

S1 Fig. Sperm isolation with the anti-PH20 IMBs and differential lysis. (A) Genotype for female epithelial cells (10^5/mL) in sample 1; (B) Genotype for sperm (10^3/mL) in sample 1; (C) Mixture of (A) and (B); (D) Sperm isolation with the anti-PH-20 IMBs for sample 1; (E) Sperm isolation with differential lysis for sample 1; (F) Sperm isolation with the anti-PH-20 IMBs for sample 2; (G) Sperm isolation with the anti-PH-20 IMBs for sample 3; (H) Sperm isolation with the anti-PH-20 IMBs for sample 4; (I) Sperm isolation with the anti-PH-20 IMBs for sample 5; (J) Sperm isolation with the anti-PH-20 IMBs for sample 6; (K) Sperm isolation with the anti-PH-20 IMBs for sample 7; (L) Sperm isolation with the anti-PH-20 IMBs for sample 8.
