# Supplementary material for: Isolating Sperm from Cell Mixtures Using Magnetic Beads Coupled with an Anti-PH-20 Antibody for Forensic DNA Analysis
Source: PLoS One. 2016 Jul 21;11(7):e0159401. doi: 10.1371/journal.pone.0159401 (PMC4956189; doi:10.1371/journal.pone.0159401)
Supplement: S2 Fig — (PDF) [file pone.0159401.s002.pdf]

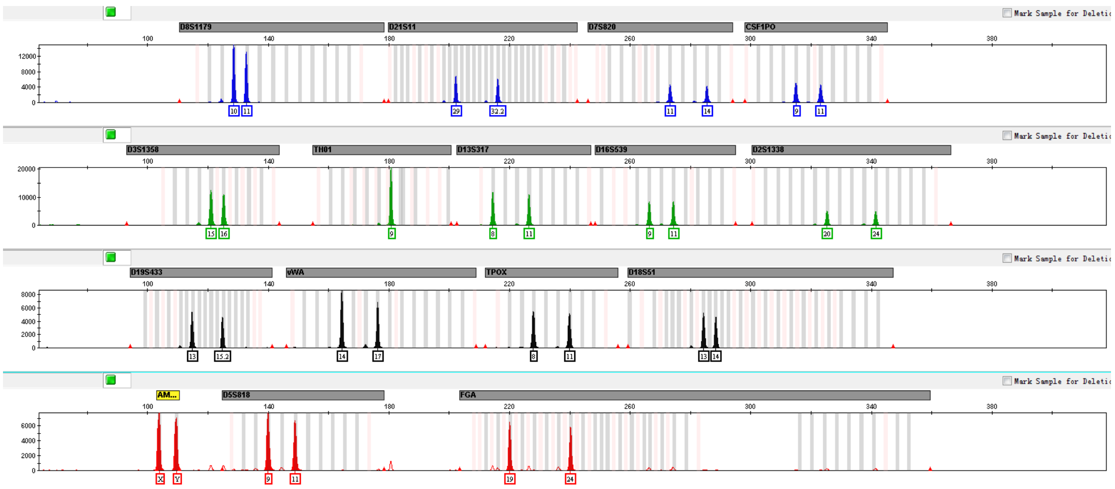


**S2 Fig. Sperm isolation from a mixture of sperm and vaginal epithelial cells using the anti-PH-20 IMBs.** Prepared mixture of sperm ($10^5$/mL) and vaginal epithelial cell ($10^5$/mL) were treated with anti-PH-20 IMBs and DNase I for sperm isolation. Isolated sperm DNA was extracted and genotyped with the AmpFℓSTR® Identifiler® Plus kit.
